# IoT-based intrusion detection system using convolution neural networks

**DOI:** 10.7717/peerj-cs.721

**Published:** 2021-09-29

**Authors:** Abdullah Aljumah

**Affiliations:** College of Computer Engineering and Sciences, Prince Sattam Bin Abdulaziz University, Alkharj, Saudi Arabia

**Keywords:** Internet of Things, Random Forest, Linear Regression, Intrusion Detection System

## Abstract

In the Information and Communication Technology age, connected objects generate massive amounts of data traffic, which enables data analysis to uncover previously hidden trends and detect unusual network-load. We identify five core design principles to consider when designing a deep learning-empowered intrusion detection system (IDS). We proposed the Temporal Convolution Neural Network (TCNN), an intelligent model for IoT-IDS that aggregates convolution neural network (CNN) and generic convolution, based on these concepts. To handle unbalanced datasets, TCNN is accumulated with synthetic minority oversampling technique with nominal continuity. It is also used in conjunction with effective feature engineering techniques like attribute transformation and reduction. The presented model is compared to two traditional machine learning algorithms, random forest (RF) and logistic regression (LR), as well as LSTM and CNN deep learning techniques, using the Bot-IoT data repository. The outcomes of the experiments depicts that TCNN maintains a strong balance of efficacy and performance. It is better as compared to other deep learning IDSs, with a multi-class traffic detection accuracy of 99.9986 percent and a training period that is very close to CNN.

## Introduction

The Internet of Things (IoT) paradigm is comprised of intelligent sensors linked by the Internet, including home appliances, phones, cars, and computers (Bhatia & Sood, 2016; Bhatia & Sood, 2017a). This form of network is becoming an increasingly important part of our daily lives, with implementations as diverse as smart grids, cities, homes, agriculture, and smart transportation (Bhatia & Sood, 2017b; Bhatia & Sood, 2019). Specifically, an IoT ecosystem is made up of web-enabled smart devices that gather, send, and act on data from their surroundings using embedded systems such as CPUs, sensors, and communication hardware. By connecting to an IoT gateway or other edge device, IoT devices may exchange sensor data that is either routed to the cloud for analysis or examined locally, Bhatia, Sood & Kaur (2019) and Bhatia & Manocha (2020). These gadgets may occasionally interact with one another and act on the information they receive (Bhatia, Sood & Manocha, 2020). Although individuals may engage with the devices to set them up, give them instructions, or retrieve data, the gadgets conduct the majority of the work without human participation  (Bhatia et al., 2020; Bhatia, Sood & Kaur, 2020).

### Research area

While the IoT will make life easier for humans, the aspect of data protection is of great concern (Thakkar & Lohiya, 2021; Mahboub, Ahmed & Saeed, 2021). IoT platform has been a popular target for cybercriminals as it faces significant risks. According to a survey from Palo Alto Networks’ Unit 42, 98 percent of intelligent system data is not encrypted, and 42 percent are having IoT sensor flaws (Gill, Saxena & Sharma, 2020; Einy, Oz & Navaei, 2021; Heal & Kunreuther, 2005). Adversaries may use the compromised computers to enter an IoT botnet and carry out effective and massive attacks. As an illustration (Antonakakis et al., 2017), the first IoT botnet was capable of exploiting compromised CCTV camcorders with generic credentials to unleash a DDoS attack over servers in October 2016. As a result of this attack, Internet service in certain areas of the United States was disrupted. Mozi, an IoT botnet discovered in April 2020, was found to be capable of launching multiple DDoS attacks. IDS have been commonly utilized for the detection of malicious network traffic to cope with the threat, particularly when security fails IoT device-end point. When threats on IoT devices become complex and stealthy, IDS must adapt to keep up with the changing security threats.

### Research gaps

IoT networks produce high-dimensional, multimodal, and temporal data due to heterogeneous structures. It is possible to uncover previously unseen trends, expose latent similarities, and obtain new insights by using big data analytics on such data. Artificial intelligence is becoming more prevalent in big data processing. Deep learning (DL) methods, in particular, have shown their ability to work with heterogeneous data. It can also analyze dynamic and large-scale data to gain information, spot data dependencies, and learn from past attack patterns to distinguish current and unknown attack patterns. Heavyweight functions like big data processing and constructing learning models must be offloaded to fog and cloud servers because IoT computers are space-restricted and have minimal storage and computing capacities. As a result, computation offloading will help minimize task execution latency and save resources in battery-powered and handheld IoT computers, but it also raises security issues. Many deep learning methods for IDS have been suggested, with some of them focusing directly on IoT (Bhatia, Sood & Kaur, 2020). However, there is still the large number of research gaps that have been identified from the previous solutions. Some of these include

1. Limited work has been done for incorporating deep learning techniques for IDS focusing on temporal aspects of the data.
2. Minimal work has been performed for heterogeneous data elements for the detection of attacks.
3. The presented approach does not incorporate the energy efficiency aspect of the IDS.
4. Even though researchers have explored predictive aspects of IDS, however, the limited focus is led on the temporal variability of the IDS.

### Fundamentals

**Figure 1:**
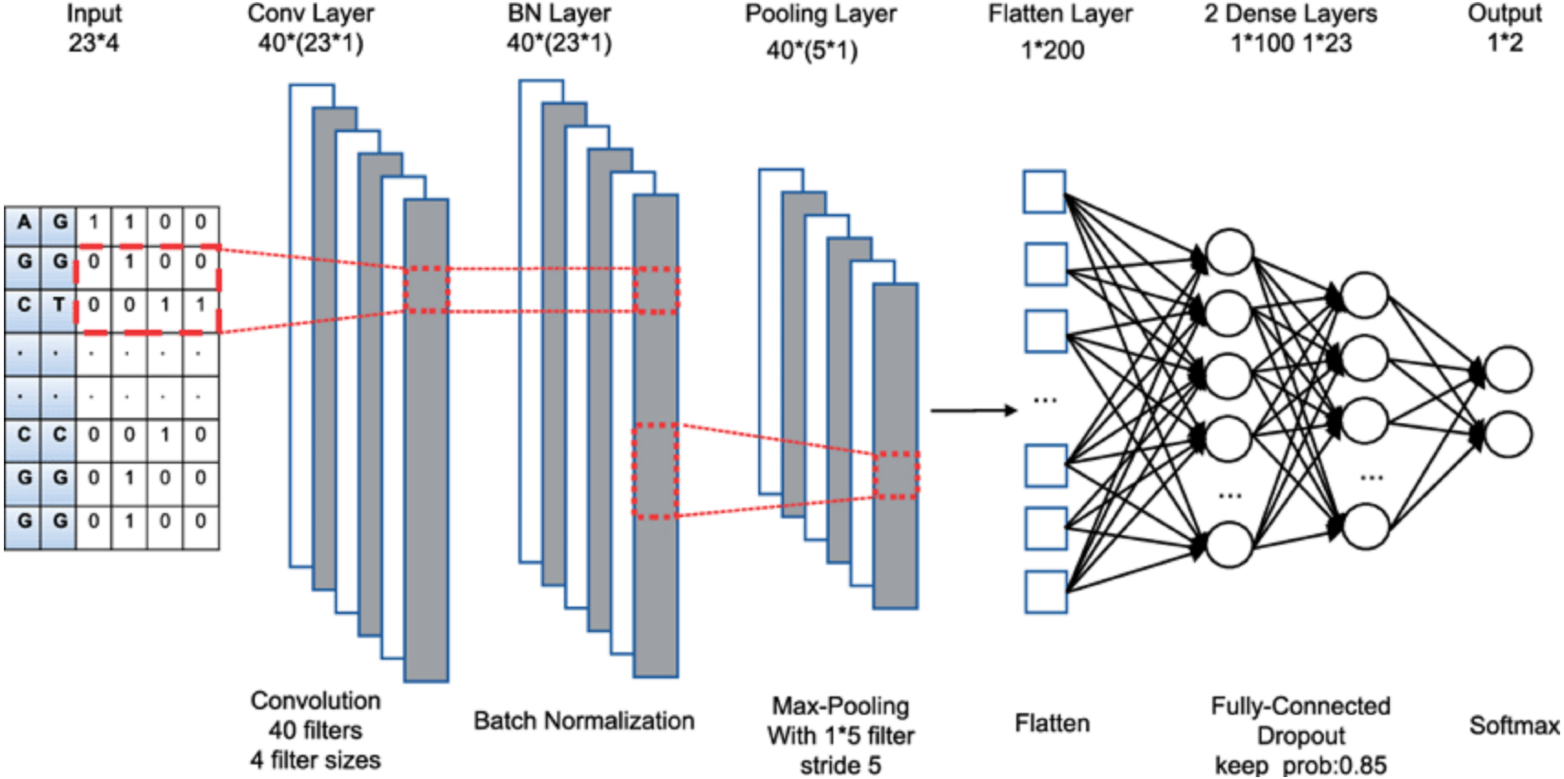
CNN architecture.

The term “deep learning” refers to the aggregation of several layers. The input layer is the first layer, which is processed to generate output *via* the final layer. In between, hidden layers are also added. Each layer is made up of a group of units known as neurons. The input layer’s size is determined by the input data’s dimension, while the output layer is made up of N nodes, which correspond to the N categories in a categorization task. Figure 1 shows a convolutional neural network with multiple layers. Below are the 3 major categories of layers:

1. *Convolutional layer*: It adds a series of filters to the input results, also known as convolutional kernels. Each filter creates a function map by sliding over the input data. The final production of the convolution layer is obtained by adding all of the generated attribute maps together.
2. *Pooling layer*: It performs subsampling over the feature maps to reduce feature reduction. The most popular pooling strategies are average pooling and maximum pooling.
3. *Fully connected layer*: This layer accumulates the outputs of the connected layers uni-formally for generating output.

### State-of-the-art contributions

The current paper suggests five architecture principles to consider when designing an accurate and efficient deep learning IDS for IoT in this article and presents TCNN, a convolutions-based version of CNN. The beneficial aspect of CNN includes minimal dependence on pre-processing, therefore decreasing the need for human effort for developing its functionalities. Moreover, it is easy to understand and fast to implement. Furthermore, it has the highest accuracy among all algorithms. Data balancing and effective function engineering were integrated with TCNN. The following are the paper’s key contributions in detail:

1. For the creation of IoT-IDS, the current paper defines 5 core design concepts, including handling over-fitting, data-set balance, function engineering, algorithm optimization, and testing on IoT dataset.
2. The current paper builds and test the Temporal Convolution Neural Network (TCNN), a DL platform to detect IoT intrusion. Convolution neural network (CNN) and causal convolution are combined in TCNN.
3. The current paper combines TCNN with synthetic minority oversampling technique-nominal continuous to address the problem of an imbalanced data-set (SMOTE-NC).
4. The current paper makes use of effective function engineering, which includes the following: (1) *Feature space reduction*: It aids in memory utilization reduction. (2) *Function transformation:* This converts warped data to a Gaussian-like distribution by applying log transformation and regular scaler to continuous numerical functions. Label-encoding, which replaces a categorical column with a special integer value, is often used on categorical features.

### Paper structure

The article structure is laid out as follows. The core architecture concepts for DL-IDS for IoTs are presented in Section 3. The next section provides an outline of similar tasks. The architecture and implementation of TCNN are described in Section 4 and 5, respectively. Section 6 brings the paper to close-by outlining prospective study directions.

## Literature Survey

Many areas in cybersecurity have used DL, involving virus identification and IDS. The current paper provide an overview of IoT-IDS networks in this section. A hybrid of recurrent neural network (RNN) and convolutional neural network (CNN), was introduced by Lopez-Martin et al. (2017) (CNN). Authors introduced layers like max-pooling, batch normalization, and dropout to cope with overfitting. To boost the model’s usefulness, authors just looked at a subset of functions. However, temproal aspects of the data is not considered for effectiveness.

On the KDD 99Cup dataset, Putchala (2017) used the Gated Recurrent Unit (GRU) algorithm. As a function filtering tool, Author used the random forest classifier. Minimizing the loss function yields the best possible output outcomes. Bidirectional Long Short-Term Memory Recurrent Neural Network (BLSTM RNN) was proposed by Roy & Cheung (2018). Authors translated categorical functions to numeric values using function normalization. The limiting aspect of the presented model is that energy effectiveness of IDS is not discussed. On the NSL-KDD dataset, Diro & Chilamkurti (2018) used a DNN model. Using stochastic gradient descent, the DNN’s loss function is minimized. Certain fog devices are in charge of DL-algorithm preparation. For updating, the local parameters are transmitted to fog-node. It encourages the right criteria to be shared and helps to prevent local overfitting. However, no aspect of prediction is discussed in the proposed model.

On the CICIDS2017 dataset, Roopak, Tian & Chambers (2019) used 4 separate categorizations of MLP, LSTM, 1 Dimensional CNN, and CNN and LSTM. The authors have duplicated documents to balance the dataset. However, no specification is presented over the balancing approach. This problem is solved by using phases like max-pooling and dropout in the model. However, the computational complexity of the presented model is very high.

The Deep Belief Network is utilized to build a deep neural network in Thamilarasu & Chawla (2019), which is then extended to an IoT simulation dataset. Each layer of the DNN model is given a cost function, which is then optimized. The limiting aspect of the presented model is that it doesnot addressees the issue of data balancing.

On the NSL-KDD data, Otoum, Liu & Nayak (2019) suggested the Stacked-Deep Polynomial Network. Authors used the Spider Monkey Optimization (SMO) algorithm (Bansal et al., 2014) to pick the best functions. The L2 regularization strategy is combined with the loss function to prevent over-fitting. However, data balancing is not discussed in the presented model.

On 2 generic data instances and the BoTIoT dataset, Ferrag & Maglaras (2019) utilized RNN with a reduced backpropagation technique. Before feeding the features to RNN-BPTT, normalization was performed.

Roopak, Tian & Chambers (2020) suggested and tested a sequential architecture that combined CNN and LSTM on the CISIDS2017 dataset. Authors used a multi-objective optimization algorithm called a non-dominated sorting genetic algorithm (NSGA) to pick the best functions. Authors used max-pooling among CNN to LSTM modules for preventing over-fitting. However, computational complexity is very high for the presented model. The BoT-IoT dataset was created by Koroniotis et al. (2019), who incorporated it to validate the prediction models. Authors used attribute normalization to scale the data within the range [0, 1] and the calculated correlation between data attributes for selection. Aldhaheri et al. (2020) suggested a DeepDCA system by incorporating Self Normalizing Neural Network and Dendritic Cell Algorithm. To choose the collection of features to feed to the BoT-IoT dataset, they used Information Gain as an attribute identification mechanism. About the fact that the authors reported findings with a balanced dataset, there is little detail about the balancing process. The author incorporated a loss function for weight modification of DNN layers for model optimization.

In the Bot-IoT dataset, Soe, Santosa & Hartanto (2019) suggested an Artificial Neural Network (ANN) for detecting DDoS vulnerabilities. Authors used the SMOTE technique to align the data set. Authors also applied function normalization to the input data before feeding it to the ANN.

On the BoT-IoT dataset, Ge et al. (2019) used feed-forward neural networks. The data instances are balanced using the algorithmic technique, rather than by oversampling, by assigning class weights to the training results. Authors modified weights using the Adam optimizer and a sparse-cross entropy loss module to refine the formula. Authors used various regularization methods, including L1, and L2 with dropout, to cope with over-fitting. Authors utilized one-hot encoding to encode categorical attributes as numerical.

Muna, Moustafa & Sitnikova (2018) suggested detecting malicious behaviors in industrial IoT using a collaborative deep autoencoder and DNN. Evaluation of loss-function, which allows weight modification and minimization of the dissimilarity between the real and expected results, yields the desired parameters.

Table 1 describes and contrasts the IDS systems in terms of the 5 techniques as described earlier. Unlike previous works, which use algorithmic-level data balancing, the current model uses the SMOTE-NC algorithm, which can accommodate both continuous and categorical functions, on the Bot-IoT dataset. To achieve successful IDS, the current paper use over-fitting and optimization strategies. For memory utilization and training time, feature space reduction is utilized along with feature transformation to achieve effective IDS.

**Table 1:** IDS for IoT.

**Reference**	**DL method**	**Over-fitting**	**Balanced data**	**Feature extraction**	**Opitmization**	**IoT dataset**
Antonakakis et al. (2017)	ANN	N	N	FN	N	Y
Putchala (2017)	GRU	N	Y	FS-RF	Y	N
Roy & Cheung (2018)	BLSTM	N	Y	FE	NY	N
Roopak, Tian & Chambers (2020)	RNN	Y	Y	FS	N	N
Diro & Chilamkurti (2018)	MLP	Y	Y	FE	N	N
Roopak, Tian & Chambers (2019)	DNN	Y	N	N	Y	N
Soe, Santosa & Hartanto (2019)	DAE	Y	N	FE	Y	Y
Otoum, Liu & Nayak (2019)	RNN	Y	Y	FS	N	N
Thamilarasu & Chawla (2019)	SDPN	N	Y	N	Y	Y
Ferrag & Maglaras (2019)	CNN	N	Y	FN	N	Y
Muna, Moustafa & Sitnikova (2018)	FNN	N	N	FSR	Y	Y
Roy & Cheung (2018)	FNN	N	SMOTE	FE	Y	Y
Koroniotis et al. (2019)	DCA	N	Y	FS	Y	Y
Ge et al. (2019)	FNN	N	Y	FSR	Y	N
THIS WORK	TCNN	Y	SMOTE-NC	FT	Y	Y

## Background Studies

DL-based IoT-IDS solutions have the goal of generating accurate models and efficient (Bhatia, Sood & Manocha, 2020). However, each model makes design decisions that can restrict its ability to achieve the goal. Some IoT DL-IDSs, for example, ignore the over-fitting issue, implements over-unbalanced data, or fail to use function engineering, all of which harm memory consumption, accuracy, and computing time. Furthermore, some IDSs do not refine the prediction model, and others are assessed using obsolete data that does not represent IoT traffic. As a result of the above findings, deep learning-specific solutions for IoT should adhere to the following aspects:

1. *Over-fitting*: It occurs when a methodology acquires a strong match on trained data but fails to generalize well to unknown data. Overfitting in deep learning can be avoided using the following techniques: (1) Using regularisation, which increases the expense of the model’s loss function for high weights. (2) Using dropout layers, which set functions to 0 and delete them at random.
2. *Data disparity*: Data imbalance corresponds to a dataset’s disproportionate allocation between groups. When a model is conditioned on an unbalanced sample, it becomes skewed, favoring the dominant groups and failing to detect the minority. The model’s effectiveness would be increased by balancing the dataset.
3. *Feature engineering*: It enables a reduction in the deep learning workflow’s memory and time costs. It also allows for optimizing the model’s accuracy by removing unnecessary features and adding feature transformation to increase the learning model’s accuracy.
4. *Model optimization*: Model optimization aims to minimize the size of a reduction function, which calculates the disparity between the expected and real outputs. This is accomplished by iteratively changing the model’s weights. The model’s effectiveness can be increased by using an optimal technique.

## Proposed Model

The proposed TCNN combines CNN architecture with causal padding to produce causal convolutions. Figure 1 displays time-series input data being subjected to 1-Dimensional general convolution having 3 kernel-size. Conspicuously, it is deduced that output is convoluted with elements of the previous layer at a specific time. As a result, the data’s temporal order is preserved, without overlapping historical data. The layers have 0 paddings with 1 kernel size to make the same length as the input to TCNN.

### Architecture in general

**Figure 2:**
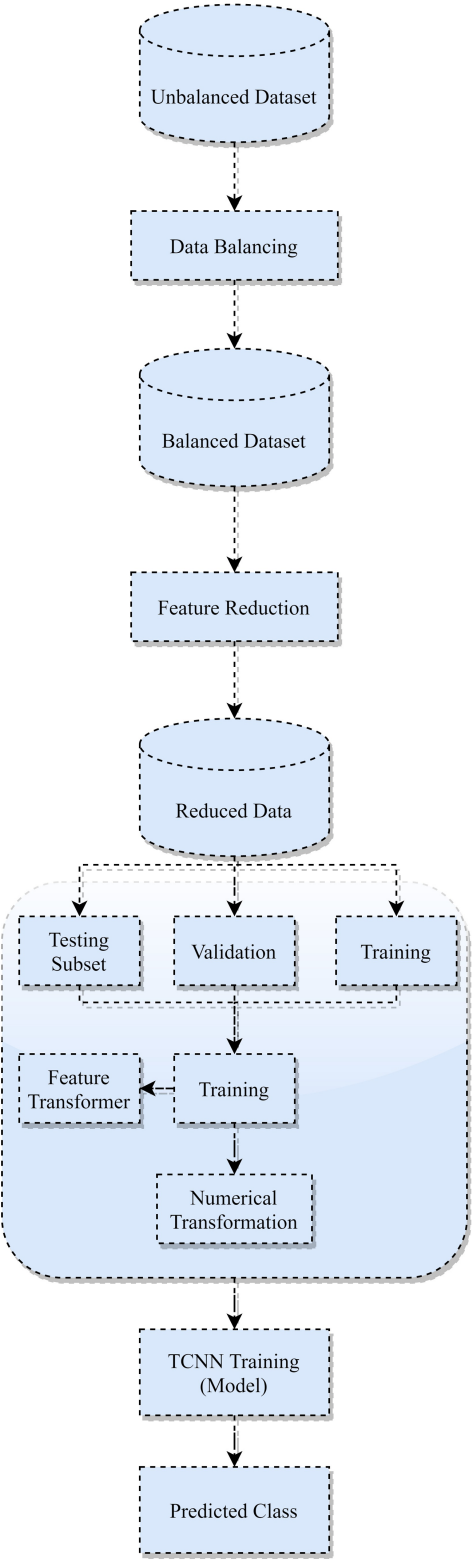
TCNN framework.

The proposed TCNN framework’s overall architecture is depicted in Fig. 2, and its implementation is outlined in Section 5. The following stages make up the proposed architecture:

1. *Dataset balancing*: An unbalanced dataset will lead to inaccurate performance. To address this problem, the current paper employs the SMOTE-NC technique to generate synthetic samples of minimal groups and can manage mixed data with categorical attributes.
2. *Initial Attribute reduction*: The current paper enhances the data instances, reducing the attribute vectors by eliminating redundant attributes and transferring memory-intensive attributes to smaller data instances.
3. *Dataset splitting:* To avoid overfitting, the dataset is divided into 3 subsets: preparation, evaluation, and checking.
4. *Second Attribute Transformation*: The current paper use attribute transformation over the trained data in this process. On the continuous numerical features, a log transformation with scaling is applied. Also, categorical attributes are encoded to replace every class column with a numerical estimate. The validation and checking subsets are then subjected to this transformation process.
5. *Trained Optimization:* TCNN model is constructed in this step, and its attributes are optimized using the Adam optimizer to validate data. It includes (i) Get gradients concerning stochastic objective at time-step t. (ii) Update biased first-moment estimate. (iii) Update biased second raw moment estimate. (iv) Compute bias-corrected first-moment estimate. (v) Compute bias-corrected second raw moment estimate.(vi) Update parameters.
6. *Categorization:* On the testing subset, the created TCNN model is used to assign every tested instance to either standard or a special category for the attack.

### TCNN model optimized training

**Figure 3:**
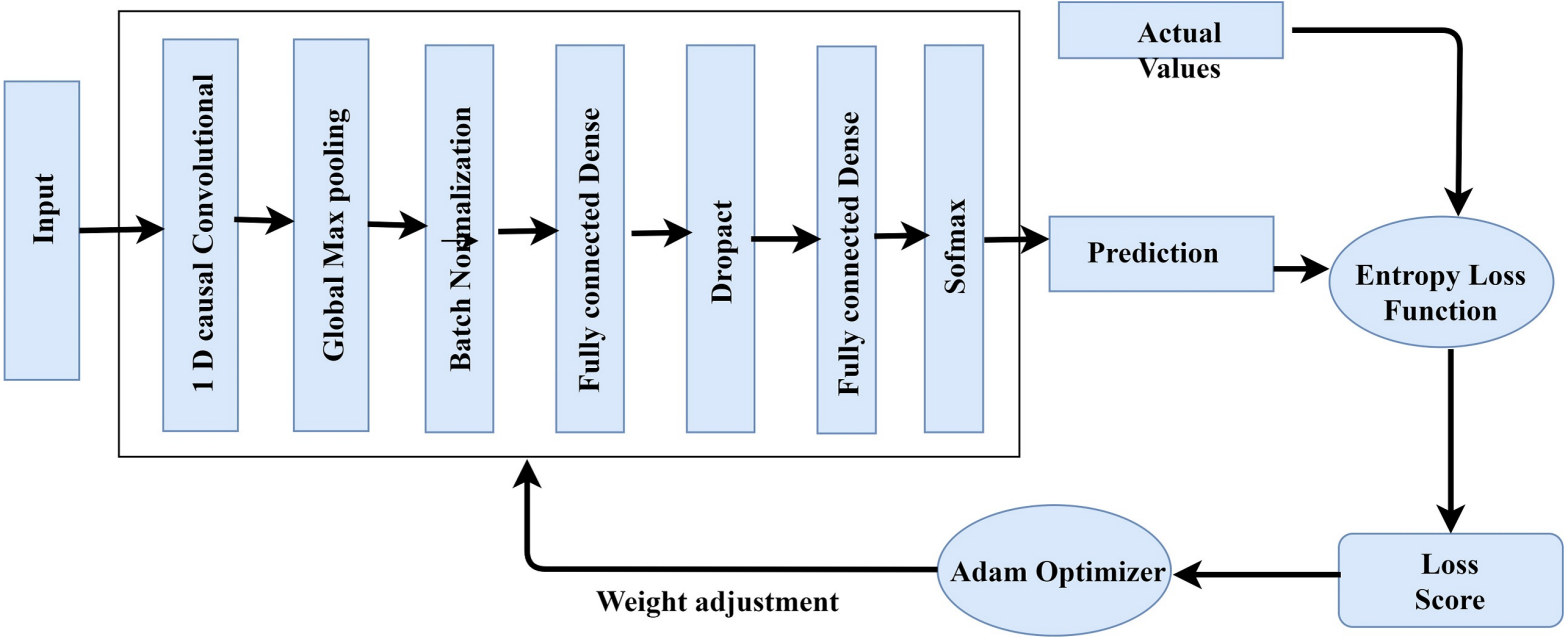
TCNN framework training.

2 1-Dimensional generic convolution layers, 2 interconnected layers, and a softmax layer for application of softmax functions make up the proposed TCNN’s training and optimization process (Fig. 3). Global maximum pooling, batch normalization, and dropout are utilized to avoid over-fitting. To change weights and refine the cross-entropy loss function, the current paper uses the Adam optimizer. It incorporates the benefits of 2 stochastic gradient descent algorithms namely Root Mean Square Propagation (RMSP) and Adaptive Gradient Algorithm. The proposed TCNN architecture’s training and optimization process are comprised of the following:

1. *Initial 1-Dimensional generic convolution layer*: with 64 filters and a filter scale of 3, it comprise the input vectors.
2. The *2^nd^ 1-Dimensional generic convolution layer* employs 130 filters with a 4 filter dimension. Until pooling, the model learns more advanced functionality in the second layer.
3. *1-Dimensional global maximum pooling layer*: This layer substitutes data that is filtered with its maximum value. By taking the highest value, it avoids over-fitting of the studied functions.
4. *Batch normalization layer*: Before moving on to the next layer, it normalizes the data from the previous layer.
5. *Fully connected dense network*: This layer has 130 intermediate nodes and a 29% dropout value.
6. *Fully connected inter-connection with soft-max activation*: It generates 5 units for multi-class grouping, 1 for each of the 5 traffic groups.

The topmost fully connected layers of the model estimate the outcome probability, as *p*(*y*|*u*_1:*n*_) = *p*(*N*_*y*_(*h*)), where N_y_ is the irregular event. The gate is modified to reflect the admission of IDS events as follows: ${i}_{t}= \frac{1}{{m}_{t}} \sigma ({W}_{i}{X}_{t}+{U}_{i}{h}_{t-1}+{b}_{i})$ where *m*_*t*_ represents the type of the event. In this proposed study, the value of *m*_*t*_ will always be equal to 1, and *σ* represents the sigmoid function of a vector. Moreover, the output can be generated as *O*_*t*_ = *σ*(*W*_*O*_*X*_*t*_ + *U*_*O*_*h*_*t*−1_ + *b*_*O*_) Based on the aforementioned steps, the computational complexity can be determined based on the number of layers (w), epochs(x), nodes at each layer(y) and input features(z). Specifically, the time complexity can be determined as O(xz ∗ ((w-1)y^2^)). In general, it is O(n^2^ ∗ (n-1)n^3^).

## Experimental implementation

Intel Quad-core i7-8449U processor has been utilized with 16 GB RAM and a 256 GB hard drive to introduce the detection learning models. To build deep learning networks, the current paper uses the Python 3.6 programming language and TensorFlow. Furthermore, Scikit-learn, Keras API, Panda, and Inmblearn are among the libraries used.

### Data instances

Bot-IoT instances is an IoT data repository launched in 2018 by the Cyber Center of New South Wales University. Legitimate and malicious traffic is created by the virtualization of intelligent systems such as smart fridges, remotely triggered garage doors, motion-controlled lights, and intelligent thermostats. The dataset contains over 73,000,000 instances, each of which is represented by 42 features. Each record is either classified as regular or as an assault. The data instances are also categorized into 4 classes including DoS, reconnaissance, DDoS, and intelligence stealing, with every class, is subdivided as shown in Tables 2 and 3.

**Table 2:** BoT-IoT attribute set.

**Attribute**	**Detail**	**Data format**
pkSeqID	Row identifier	Integer
stime	Record start time	Float
flgs	Flow state flags seen in transactions	Category
proto	Textual representation of transaction protocols presents in network flow	Category
Sport	Source port number	Category
Dport	Destination port number	Category
Pkts	Total count of packets in transaction	Integer
State	Transaction state	Category
Dur	Record total duration	Float
Mean	Average duration of aggregated records	Float
Dpkts	Destination-to-source packet count	Integer
Sbytes	Source-to-destination byte count	Integer
Dbytes	Destination-to-source byte count	Integer
TnBPSrcIP	Total number of bytes per source IP	Integer
TnP_PerProto	Total number of packets per protocol	Integer
AR_P_Proto_P_SrcIP	Average rate per protocol per source IP.	Float
AR_P_Proto_P_Dport	Average rate per protocol per dport	Float
Pkts_P_State_P_Protocol_P_SrcIP	Number of packets grouped by state of flows and protocols per source IP	Integer
Srate	Source-to-destination packets per second	Float

**Table 3:** BoT-IoT data set.

Attack	Category	Total records
Attack	Reconnaissance	1,593,444
Attack	DoS	254,659
Attack	DDoS	12,123,565
Attack	Information theft	1,654
Normal	No	9,658

### Dataset balancing

Dataset balancing is the process of balancing several datasets. There are 9,429 standard samples and 73,000,000 vulnerable instances in the repository. There are 489 regular samples and 3,459,125 vulnerable instances. More than 98 percent of the instances fall into the DDoS and DoS groups. As a result, the learning algorithm will correctly estimate the majority classes but miss the minority classes, indicating that the model is biased. To address this problem, various re-sampling techniques are presented, including random oversampling, which replicates the same samples of minority classes at random, and oversampling by creating synthetic samples of minority classes using techniques including synthetic minority oversampling technique, and synthetic minority oversampling technique. The current paper uses the SMOTE-NC methodology in this study because it can handle mixed data instances with continuous attributes. In the training subset, the minority groups, such as common and stealing, are increased to 99,98,989 samples.

### Attribute minimization

The vital goal is to create an effective IDS for use in the IoT environment. As a result, it is critical to increasing the detection models’ performance by attribute reduction, as well as memory consumption optimization with minimized computing complexity. A total of 2.9 GB of memory is used while all of the functions are used. Feature space reduction reduces computational overhead while still speeding up preparation and identification. The following steps are added to the dataset, and the memory usage is successfully reduced to 668 MB, a 77 percent reduction.

1. *Data type conversion to categorical class*: The number of attributes and corresponding data formats are encoded for each form. The memory-intensive features flags, proto, saddr, sport, daddr, dport, state, category, and subcategory are encoded as elements. Object attributes are translated to category datatype since it is more powerful.
2. *Int64 to Int32 data type conversion*: The dataset’s integer properties, as depicted in Tables 4 and 5, are accumulated in the Int64 (8-byte) data types by default. These features do not surpass the capability of the Int32 (4-byte) sort, according to current investigation. As a result, all the values of the Int64 class are converted into the Int32 type, which consumes 50% storage as compared to the Int64 type.
3. *Removing superfluous features*: The current paper removed certain superfluous features from the dataset, such as (1) “pkSeqID”: it serves the same purpose as the index that is created automatically. (2) “stime” and “ltime” are acquired in the “dur” function for calculating the length from “stime” to “ltime”.

### Transformation of attribute

**Figure 4:**
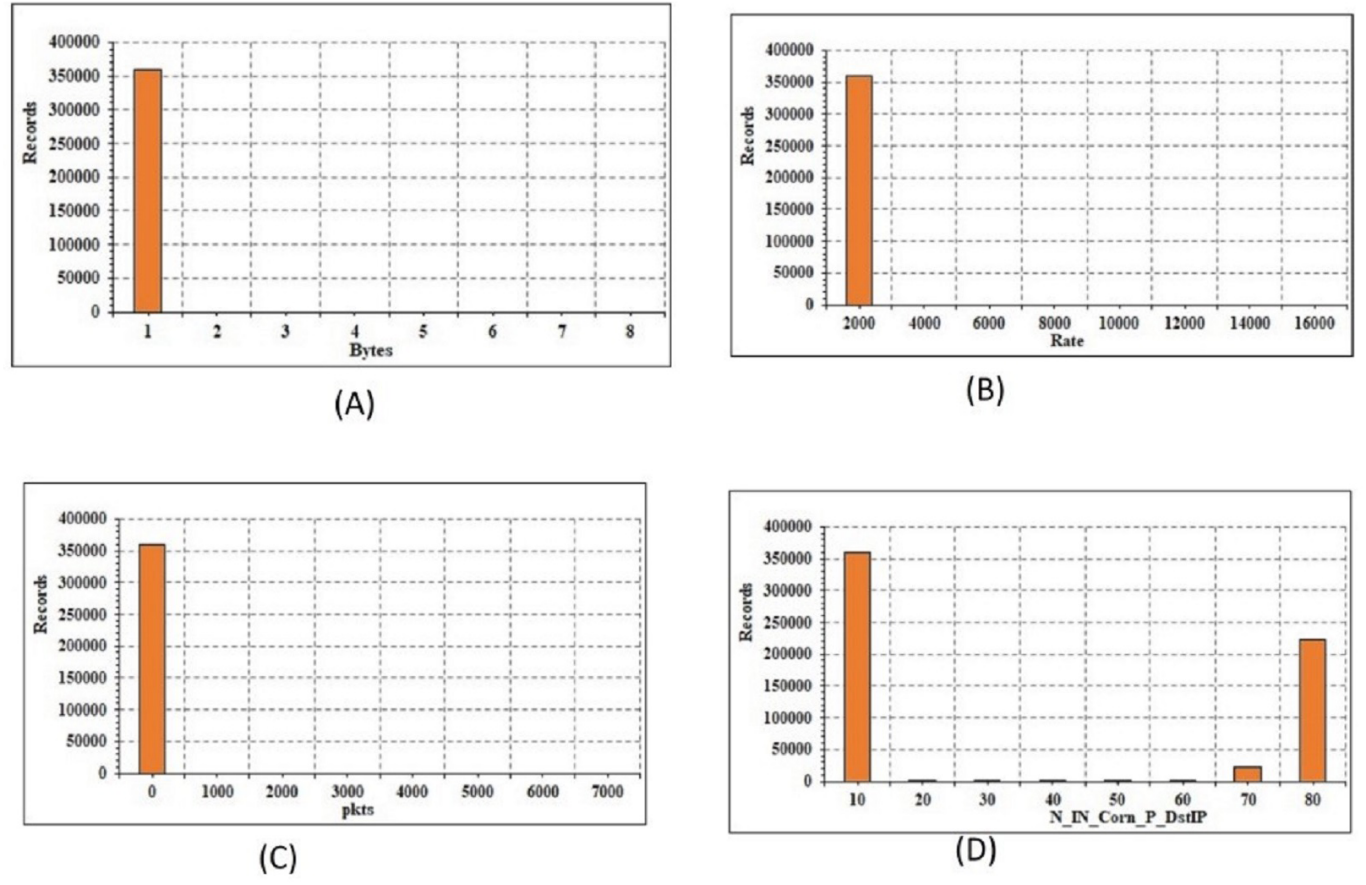
Energy conservation analysis: (A) records *vs* bytes; (B) records *vs* rate; (C) records *vs* packet size; (D) records *vs* IP.

The current paper define the transformation of mathematical and categorical elements. The transition is only extended to the training subset after the data instances have been broken for train, validation, and testing subsets. The validation and testing subsets are then subjected to the same transformation. *Transformation of numerical features:* The data includes 32 mathematical attributes, both specific and seamless estimates. There are 2 distinct functions, namely “spkts” and “dpkts”, each of which has a finite number of values. As a result, no feature engineering is needed. The graphical structure of the 4 attributes is shown in Fig. 4. The continuous attributes are not necessarily distributed, which typically affects the output of linear models. To do this, the log-based modification and regular scaler are added to the seamless attributes to achieve a Gaussian distribution:

1. *Log transformation*: The current feature value y = log_10_y, where y is the attributes original value.
2. *Standard scaler*: This function calculates the average and standard deviation of a range. The characteristics are then transformed into a Gaussian distribution.

### Splitting a dataset

The most popular methods for separating data sets are traditional splitting and cross-validation. It is primarily utilized in machine learning to avoid over-fitting. Cross-validation raises the training cost when a massive data repository is utilized for deep learning. The data-set is divided into 3 subsets in this study, using the traditional 3-way split: preparation, evaluation, and research. Also, where over-fitting occurs, regularization is used to deal with it. A stratified split is often utilized to guarantee that each split contains a part of each category.

**Table 4:** Training dataset.

DDoS	155,151,156	DDoS	145,165,189
DoS	75956	Normal	121200
Normal	236	Theft	123213
Theft	59	Reconnaissance	72356

**Table 5:** Types of attributes.

Type	INT64	Float-64	Object
Attributes	23	16	10

### Deep learning techniques

TensorFlow with Keras API is used to build all deep learning models. Preprocessing, templates, layers, and optimizers are among the Keras programs included. In both versions, the same activation functions are used. The ReLu activation function is used to analyze cognitive interactions between given input and output. Softmax is utilized as the output activation module, and a generalized logistic regression-based activation function is used as the input layer activation function. In softmax, the frequency of production units is equal to the number of attack groups plus the standard class. Table 6 depicts the deep learning architectures of TCNN as well as hyperparameters. Overfitting is addressed using methods such as global limit pooling, batch normalization, and dropout. The Adam optimizer is chosen to change the weights because it outperforms other optimizers such as SGD and AdaGrad.

**Table 6:** Hyper-parameters of DL model.

Parameter	Hyper-parameter	Value	Activation function
TCNN	1st Convolution Layer	Filter=64, size=3	ReLU
TCNN	2nd Convolution Layer	Filter=128, size=3	ReLU
TCNN	Fully Connected	neurons=128, dropout=0.3	ReLU
TCNN	Fully Connected	neurons=5	Softmax
Optimizer	–	ADAM	–
Batch	–	2048	–
Epochs	–	16	–

### Assessment

The current paper equates TCNN’s output to that of 5 machine/deep learning algorithms of Random Forest (RF), Long Short Term Memory (LSTM), Convolution Neural Network (CNN), Decision Tree (DT), and Logistic Regression (LR).

### Metrics for performance estimation

The following metrics are used to test the multiclass detection models:

1. *Effective Measure*: In this, assessment is done on how well the identification model distinguishes between various types of network traffic. The current paper use the following metrics to do this: (i) Precision = TP/TP + FP + FN (ii) Accuracy = TP + TN/TP + TN + FP + FN (iii) F1 score = 2 ∗Precision Recall/Precision + Recall (iv) Recall = TP/TP + FN where TP, TN, FP, and FN represent true positives, true negatives, false positives, and false negatives, respectively.
2. *Log loss*: It assesses the accuracy of a categorization technique with a probabilistic measure as an output. The log loss would be 0 in a perfect model, and it would increase as the expected likelihood differed from the real label.
3. *Training time*: this is the amount of time it takes to construct a classification technique.

### Prediction evaluation

Table 7 shows the effects of evaluating RF and LR on initial and re-balanced data-sets. All of the experiments had almost identical training and testing ratings, indicating that there is no over-fitting. In terms of LR, SMOTE-NC oversampling improves accuracy, recall, and F1-score, implying that it is better at identifying minority groups. Oversampling, on the other hand, has little bearing on the RF’s efficacy.

**Table 7:** Performance analysis.

**Prediction model**	**Oversampling**	**Phase**	**Log loss**	**Accuracy**	**Precision**	**Recall**	** F1-score**	**Training time**
LR	None	Training	0.0569	97.2365	59.6598	81.2654	52.12654	503
LR	None	Testing	0.0568	97.6598	59.6589	82.2656	52.1458	
LR	SMOTE-NC	Training	0.07458	99.659	75.2659	99.659	79.2658	710
LR	SMOTE-NC	Testing	0.7776	99.2659	74.2659	98.659	78.965487	
RF	None	Training	0.20001	97.5896	80.2654	98.5659	86.21547	189
RF	None	Testing	0.19546	97.4584	77.2154	98.888	84.2655	
RF	SMOTE-NC	Training	0.19595	96.63265	79.6598	98.1547	86.2654	201
RF	SMOTE-NC	Testing	0.19478	96.2565	75.3265	98.2658	82.658	
DT	None	Training	0.22001	96.4896	78.2654	97.4659	84.21547	195
DT	None	Testing	0.22546	96.8584	74.5154	97.788	83.5655	
DT	SMOTE-NC	Training	0.23595	94.65265	76.9598	92.6547	82.2654	251
DT	SMOTE-NC	Testing	0.29478	93.1565	72.6265	97.2658	81.658	
CNN	None	Training	0.21001	96.5896	79.2654	97.5659	85.21547	203
CNN	None	Testing	0.21546	96.1584	75.1154	97.488	83.1655	
CNN	SMOTE-NC	Training	0.21595	95.8265	77.4598	97.7847	85.564	285
CNN	SMOTE-NC	Testing	0.20478	95.45565	73.2265	97.9658	80.658	
LSTM	None	Training	0.25201	97.4125	79.1256	97.1236	85.20124	232
LSTM	None	Testing	0.20125	94.2666	76.6595	96.8528	83.2555	
LSTM	SMOTE-NC	Training	0.202325	95.55265	78.6298	94.5547	85.2554	212
LSTM	SMOTE-NC	Testing	0.20232	95.1455	74.36665	97.6658	81.558	

### Deep learning model evaluation

To achieve the best efficiency, The current paper runs a sequence of experiments with various hyper-parameter measures (as an instance batch size, learning rate, number of units, and number of layers in each layer). The optimizer learning rates are investigated. When the learning rate is 0.001, the best results are obtained. Also measured are various epoch counts (10, 15, 25, 55, and 100) as well as different batch sizes of 105, 512, 1024, and 2048. The current paper can see that elevation of the frequency of epochs causes the learning mechanism to slow down. Similarly, reducing the batch size has little effect on results. The batch size and number of epochs for TCNN are set to 25 and 2048. Table 8 depicts the TCNN’s multi-class classification accuracy and log failure during the preparation and testing processes. TCNN achieves enhanced efficiency in the first epochs, implying that 15 epochs are sufficient. Furthermore, there is no evidence of overfitting in the training and validation findings. Table 7 depicts the LSTM and CNN log failure data. In terms of log loss, The current paper can see that TCNN is better than LSTM and CNN. As some accuracy outcomes surpass 99.99 percent, the current paper can see that DL models outperform RF and LR. The precision findings are very similar, but in terms of efficacy measures, TCNN marginally outperforms LSTM and CNN. It can be depicted that DL models do well even though data balancing is not used. It is observed that a small and very minor reduction of efficacy for LSTM and TCNN when SMOTE-NC oversampling is used. The efficacy of CNN, on the other hand, improves marginally by using SMOTE-NC oversampling. CNN, therefore, takes less time to practice than TCNN and LSTM. Since it is the nearest rival to CNN in terms of preparation time and accuracy, TCNN has a fair trade-off between efficacy and performance. Moreover, the energy conservation analysis is depicted in Fig. 4 for the proposed model.

**Table 8:** Performance analysis.

Oversampling	Log-Loss	Accurate rate	Precise-Rate	Recall	F1 measure	Training-Delay
None	0.00124	99.8956	99.2625	96.2154	98.6598	419
SMOTE-NC	0.1212	99.2356	97.1458	94.6598	95.1457	457

### Evaluation of related work

Table compares the success of the current model to that of related approaches validated on the Bot-IoT data. In terms of precision, accuracy, F1-score, recall, training time, and categorization task, the distinction is made.

1. *Binary classification task:* distinguishing between standard and attack records is the aim of this task.
2. *Classification task for standard/one-attack records:* This task attempts to differentiate between normal records and specific-attack.
3. *Multi-class categorization:* It attempts to assign a record to 1 of the 5 classes (1 regular class and 4 attack classes) under which it belongs.

Multi-class categorization is considered to be the most difficult task, while normal/1-attack classification is the simplest since the data only includes 1 form of attack, resulting in less diversity and easier learning for the detection model. It can be seen from Table 8 that the presented model has a 99.9% effectiveness rate. This finding, on the other hand, can be explained by the fact that the presented technique is designed to differentiate between regular traffic and only 1 form of attack, namely DDoS. Since they are tested under a multi-class categorization task, the 3 DL models LSTM, TCNN, and CNN outperform other techniques. In terms of training time, it can be seen that LSTM, TCNN, and CNN provide optimal results. This is due to the use of simplistic DL algorithms with greater batch sizes and fewer layers, as well as feature engineering that decreases computation complexity.

## Conclusion

In this article, the current paper presents five design principles for developing a DL-based IDS framework for the IoT environment that is both accurate and powerful. The current paper modeled and implemented the Temporal Convolution Neural Network (TCNN), which incorporates convolution neural network (CNN). SMOTE-NC data balancing and efficient attribute engineering, which includes attribute reduction and transformation, are combined with TCNN. On the Bot-IoT dataset, TCNN was tested and compared to logistic regression, random forest, decision tree, LSTM, and CNN. The findings of the evaluation suggest that TCNN maintains a successful balance of efficacy and performance. It outperforms state-of-the-art DL IDS models, which were validated using the current data repository, with a multi-class traffic detection accuracy of 99.9986 percent. Overall, an enhancement of 2.56% was realized in comparison to the other approaches. In terms of training time, it also performs very similarly to CNN. Another architecture theory worth considering in future work is measuring the reliability of IDS against severe threats, for assessing the DL model and cause prediction inaccuracy.

##  Supplemental Information

10.7717/peerj-cs.721/supp-1 Supplemental Information 1 All Source Files

## Additional Information and Declarations

### Data Availability

The following information was supplied regarding data availability: The dataset is available at IEEE Dataport: Nour Moustafa, October 16, 2019, “The Bot-IoT dataset”, IEEE Dataport, doi: https://dx.doi.org/10.21227/r7v2-x988. Neural Designer was used in this research and is available at: https://www.neuraldesigner.com/.
